# Demand for Pharmacogenomics and Personalized Medicine in the United Arab Emirates

**DOI:** 10.3390/jpm12010104

**Published:** 2022-01-14

**Authors:** Yazun Jarrar, Su-Jun Lee

**Affiliations:** 1 Department of Pharmacy, Al-Zaytoonah University of Jordan, Amman 11733, Jordan; yazun.jarrar@zuj.edu.jo; 2 Department of Pharmacology and Pharmacogenomics Research Center, College of Medicine, Inje University, Busan 50834, Korea

The application of personalized medicine (PM) is rapidly evolving. Now extending beyond simple genomics, PM is applied to make medical decisions by integrating daily health, omics, disease characteristic, organ function, environmental, and lifetime exposure data. Pharmacogenomics, a key field in the study of PM, is a fusion of pharmacology and genomics, and its research mainly investigates the pharmacological properties of drugs and genes related to drug absorption (A), distribution (D), metabolism (M), and excretion (E) (collectively, ADME), as well as drug effects. Inappropriate drug selection and/or dosing is a major reason for hospitalization [1]. Some patients may not respond to drug therapy or suffer from toxic effects more than others [2,3]. Pharmacogenomic testing aims to personalize pharmacotherapy and reduce toxic effects caused by abnormal drug ADME or gene mutations that directly affect drug responses [4]. Recognizing the importance of genomic medicine and pharmacogenomic testing in PM, the government of the United Arab Emirates (UAE) recently began to support genomic projects, including the Emirati Genome Program genomics and pharmacogenomics research, and the Department of Genetics and Genomics of the College of Medicine and Health Sciences at the University of Emirates began to provide graduate certificates and perform research in pharmacogenomics. Pharmacogenomics-related research articles published in the UAE began to appear in the National Center for Biotechnology Information (NCBI) database in 2019 and have increased significantly in number during the past two years [5]. Most pharmacogenomics research published in the UAE has focused on the current knowledge and perception of pharmacogenomic testing among healthcare students and workers, as well as mapping future pharmacogenomics education [6,7,8,9].

In order to carry out pharmacogenomic testing in clinical practice in the UAE, the knowledge of current medical experts, the awareness of stakeholders, and the determination of which drug to try should be considered first. The current knowledge, perception, and practice of genomic medicine and pharmacogenomic testing among pharmacists were assessed in a recent article in the *Journal of Personalized Medicine*, entitled “Genomics and pharmacogenomics knowledge, attitude and practice of pharmacists working in United Arab Emirates: Findings from focus group discussions—a qualitative study” [6]. This study was based on a survey of pharmacists, who play a major role in personalized pharmacotherapy, following invited discussion sessions at the College of Medicine and Health Sciences at the University of Emirates to ensure attendance and accurate extraction of information from participants. The authors found that the participating pharmacists had a poor grasp of pharmacogenomic testing; most participants had not heard of pharmacogenomics and had not studied it in university courses. Most participants were also unaware that laboratories provided pharmacogenomic testing or the applications of pharmacogenomic testing in clinical practice. This poor knowledge of pharmacogenomics resulted in a sense of powerlessness in decision making and drug therapy counseling among most participants. However, the participants had a highly positive attitude toward pharmacogenomic testing. The authors of the study indicated that religious and cultural issues may create obstacles to the implementation of medical genomics in the UAE. These factors may have been overestimated by the researchers because medical genetic testing is currently applied in Middle Eastern countries, including the UAE, against diseases such as thalassemia, cancer, and cystic fibrosis, and religion and culture have not been reported to negatively impact genomic testing in these medical fields. One major advantage of the design of the survey-based study was that the participants were pharmacists who were in direct contact with actual patients [6]. Although most participants had poor knowledge of pharmacogenomics, the pharmacists emphasized the importance of including more courses and lectures on this topic during university education and holding related workshops and conferences in the UAE.

Subsequently, multiple studies have been conducted by a UAE-based research group on participants’ knowledge of and attitude toward pharmacogenomics [7,8,9]. This group found that healthcare students have poor knowledge but a highly positive attitude toward pharmacogenomic testing [7], with most students responding that a lack of pharmacogenomics knowledge represents a major obstacle to the implementation of pharmacogenomic testing in clinical practice. This finding emphasizes the importance of adding pharmacogenomics lectures to university syllabi for the education of healthcare providers in the UAE. In another study, this research group reported that other healthcare providers such as physicians have a strong interest in pharmacogenomics but lack confidence in their genetic knowledge to select appropriate drugs and doses based on genetic analysis data [8]. Most healthcare providers responded that they require additional workshops, lectures, and training on pharmacogenomic testing and its application in clinical practice. Thus, awareness of pharmacogenomics should be increased among healthcare students and providers. In a third study, this research group evaluated the interest of powerful UAE stakeholders in applying pharmacogenomic testing [9]. Although the interviewed stakeholders showed an interest in pharmacogenomic testing, they responded that the demand for such testing is not high in clinical practice at present. The reason for this response may be a lack of awareness about adverse drug reactions or toxicity caused by genetic factors. Some stakeholders also expressed concerns about the cost effectiveness of pharmacogenomic testing and the legal aspects of genomic medicine. All of these barriers against the implementation of pharmacogenomic testing in clinical practice are the result of a lack of basic research and clinical evidence supporting the use of pharmacogenomic testing in the UAE. For pharmacogenomic testing to be widely accepted in the UAE, it is necessary to conduct many clinical and basic research studies to provide evidence of the correlation between drug side effects and genetic variation. The reporting of such research findings by the media would greatly increase the demand for pharmacogenomic testing by government officials and the public.

These findings among healthcare providers in the UAE are consistent with those in other Middle Eastern countries. Similar results were reported in Jordan [10], the West Bank of Palestine [11], Egypt [12], and Qatar [13], where all participating pharmacists had poor knowledge but highly positive attitudes regarding pharmacogenomic testing. These findings indicate that there is a demand to improve knowledge about pharmacogenomic testing among pharmacists and other healthcare providers in Middle Eastern Arabic countries. These surveys of the attitudes toward and knowledge and practice of pharmacogenomic testing in Middle Eastern countries were conducted recently, and pharmacogenomic testing is still not implemented in clinical practice in these countries. The lack of pharmacogenomics knowledge and training appears to be the major obstacle to the implementation of pharmacogenomic testing in clinical practice in the Middle East, including the UAE [6,11,12,14]. Some universities in the UAE and Qatar have recently established science departments providing graduate certificates in pharmacogenomics and PM. Lectures based on pharmacogenomics knowledge base (PharmGKB) guidelines [15] were also recently introduced in medical and pharmacological courses in some Middle Eastern universities, and workshops on the clinical implementation of pharmacogenomics have been held in the UAE, Egypt, and Jordan [16]. 

Numerous published cases have described reduced drug side effects and improved drug efficacy following the clinical application of pharmacogenomic testing. With the increasing availability of continuous genomics and clinical data and the development of supercomputer modeling technology, PM application is becoming more sophisticated. As PM technology advances, the knowledge and education of pharmacists working in clinical settings is becoming more important and will play a key role in the further development of PM. As recommended in focus group discussions from one study in the UAE [6], the realization of PM in the UAE requires university education in pharmacogenomics, more inter-university workshops, a central role for pharmacists in PM, and the construction of an electronic decision-making system. In particular, the improvement in pharmacists’ knowledge and experience in pharmacogenomics will lead to various beneficial events in PM clinical application, including improved patient understanding of the role of genomics in pharmacotherapy, reduced medical costs, improved quality of life, and effective communication between patients and doctors.

## Data Availability

Not applicable.
